# A New Method for Constructing a Base for Artificial Teeth

**Published:** 1853-10

**Authors:** 


*A New Method for Constructing a Base for Artificial Teeth.*—Dr. A. Fitzpatrick, dentist, of Calcutta, has recently published a small treatise on the application of artificial teeth, in which he describes a new method he has adopted for the construction of the base. The object which he proposes to gain by it is, to obviate the inconvenience arising from having the whole surface of the palate covered with a plate. To prevent which, large holes are cut in the plate, leaving those parts of the palate which are supplied with sensation from the anterior and posterior palatine nerves, uncovered. The author has applied several dental substitutes, mounted on bases of this description, and with such good results as to induce him to recommend their use to the profession. One of the editors has applied an upper set of teeth, mounted on a base of this kind, though in a somewhat modified form, which have been worn about ten weeks, with great comfort and satisfaction. Plates constructed in this way, should be slightly thicker than those ordinarily employed, to prevent the liability of their being too easily bent. When fitted with the necessary accuracy, they adhere with sufficient tenacity, and are certainly worn with less inconvenience than dental substitutes as usually constructed, but whether the base will retain its adaptation as long, is a question which can only be determined by experience. Dr. Fitzpatrick is sanguine in the belief that it will ultimately supersede the base at present employed.

